# Country of qualification is linked to doctors’ General Medical Council performance assessment rate, but is it linked to their clinical competence?

**DOI:** 10.1186/s12916-017-0918-1

**Published:** 2017-08-07

**Authors:** Richard Wakeford

**Affiliations:** Hughes Hall, University of Cambridge, Cambridge, CB1 2EW UK

**Keywords:** Revalidation, Assessment of competence, Clinical assessment, International graduates, Underperformance

## Abstract

Mehdizah and colleagues recently described the prevalence of General Medical Council regulatory performance assessments by doctors’ country of primary medical qualification. This article has caused anger within the UK–international medical community because it identifies graduates of certain countries with significantly raised prevalence.

The present article comments on evidence from published Royal College of General Practitioners’ data that support these conclusions. However, in an increasingly international age of medical education, the ambiguity of attributions of qualifying from a certain country needs addressing. Some medical students of British nationality, for example, who fail to obtain a place at a UK medical school, train in medical schools abroad, and thus may be identified as international medical graduates.

Please see related article: https://bmcmededuc.biomedcentral.com/articles/10.1186/s12909-017-0903-6.

## Background

In the 1970s, significant numbers of doctors who were trained outside the UK joined the National Health Service (NHS). The first tests to assess overseas-qualified doctors were introduced by the General Medical Council (GMC) in 1975. Originally known as ‘TRAB’ (Temporary Registration Assessment Board) tests, these were later renamed ‘PLAB’ (Professional and Linguistic Assessments Board) tests [1]. There has been interest in their clinical performance as assessed by postgraduate examinations (e.g. [2, 3]), and their experiences of disciplinary attention by the GMC. [4].

When the GMC has concerns about a doctor’s performance, it may, as part of its regulatory *armamentarium*, require him or her to undergo a ‘performance assessment’; a set of assessments tailored to the individual’s level and type of practice. After summarising previous evidence on differential performance, a recent paper by Mehdizadeh et al. [5] analyses new GMC data on doctors who received a GMC performance assessment (PA) between 1996 and 2013 by demographic variables, including country of primary medical qualification (PMQ).

### Findings of the study by Mehdizadeh *et al.*

Highly significant differences were found between the various countries of PMQ, with an incident rate ratio (IRR) of doctors from Bangladesh 13 times higher the UK baseline. Egyptian and Nigerian-trained doctors had an IRR of 8; and for doctors trained in countries acceding to the EU in 2004, the IRR was 4 times that for UK doctors.

However, the authors note that very small actual numbers are represented by these statistics. The reasons for the differences are unclear and need further investigation. They might derive from true differences in competency and training standards, or from differences in the way(s) this group of doctors is treated both by society and employers.

### Credibility of the findings: triangulation?

The publication of Mehdizadeh et al.’s data caused understandable dismay among graduates of countries with high IRRs. Within two weeks of the paper’s online publication, a news report appeared in the BMJ [6] in which the president of the British Association of Physicians of Indian Origin (BAPIO) was quoted as saying that the paper was “useless really, it doesn’t mean anything…it is retrospective.” The president also asserted, “it doesn’t say anything about the competence of these doctors.” In a sense, this is correct, as the outcome of the PA was not considered, only the PA referral; however, there must be serious concerns about a doctor’s competence for a referral to have been made.

Is any evidence available that could be used to triangulate the implications of this paper – that those groups with high IRRs and most likely to be called to PAs are clinically less competent? The GMC publishes extensive data on UK medical graduates, and their performance in postgraduate examinations and in Annual Reviews of Competency Progression [7], but does not yet differentiate between PMQ countries. However, the Royal College of General Practitioners does so in its annual statistical report and has done since 2008 [8]. Table 1 contrasts Clinical Skills Assessment (CSA) results in the last year for which data are available (2015–2016) [9] with the data presented in the Mehdizadeh et al. paper for the 10 countries common to both lists.

**Table 1:** Performance assessments by country of primary medical qualification versus MRCGP Clinical Skills Assessment performance

Doctors’ country of PMQ	No. of doctors on LRMP 2013	No. of PAs 1996–2013	Crude rate of PAs/1000 doctors	IRR^*^	No. of first-attempt candidates taking CSA 2015–16	Mean CSA scaled mark 2015–16 (pass = 0)	CSA fail rate % 2015–16	AKT fail rate % 2015–16
A	B	C	D	E	F	G	H	I
Bangladesh	874	23	26.32	13	9	–11.78	88.9	71.4
Egypt	3215	45	14.00	8	10	–6.20	80.0	63.6
India	25114	242	9.64	5	90	–3.12	53.3	50.0
Iraq	2326	30	12.90	8	10	–1.00	50.0	50.0
Ireland	4020	28	6.97	2	13	9.77	15.4	8.3
Nigeria	4067	47	11.56	8	71	–4.85	67.6	47.6
Pakistan	9400	61	6.49	4	108	–4.67	68.5	51.7
South Africa	5444	17	3.12	1	7	5.29	28.6	n/a^#^
Sri Lanka	2376	21	8.84	4	8	–12.50	75.0	0
UK	164691	332	2.02	1	2572	11.20	10.8	14.6

*AKT* applied knowledge test, *CSA* Clinical Skills Assessment, *IRR* incidence rate ratio, *LRMP* list of registered medical practitioners, *MRCGP* Membership of the Royal College of General Practitioners, *PA* performance assessment, *PMQ* primary medical qualification

^*^IRR is estimated from Figure 7 in Mehdizadeh et al. [5]; # AKT fail rate not reported

Calculated from the data in Table 1, the IRR for the 10 PMQ countries common to this paper’s data and those listed in the MRCGP (Membership of the Royal College of General Practitioners) report correlates strongly and negatively with the mean CSA scores (columns E and G; *rho* = –0.66, *P* < 0.05), and positively with the associated fail rates (columns E and H; *rho* = 0.73, *P* < 0.02). Similar patterns result from correlating the crude PA rates (column D) with the CSA scores (*rho* = –0.65, *P* < 0.05) and fail rates (*rho* = 0.71, *P* < 0.05); results of the multiple-choice MRCGP applied knowledge test (e.g. *rho* IRR to AKT fail rate, columns E and I = 0.70, *P* < 0.05); and in RCGP data from the previous year [10].

Differential performance in the MRCGP assessments by country of PMQ parallels that reported in this paper. The credibility of the order of the presumed clinical competence of doctors from the various countries of PMQ is thus supported; however, as suggested by a court action in 2014 [11], examination performance may not accurately reflect clinical competence.

### The problem of expatriate medical students

‘Country of medical training’ does not always equate to ‘country of origin’ or ‘nationality’. UK nationals can train elsewhere, emerging as an ‘International Medical Graduate’ (IMG). Thus IMGs may be British, and it should not be assumed that someone with a qualification from (for example) Cluj Napoca is Romanian.

In GMC research towards a paper on PLAB tests [2], we noted that graduates from a variety of countries (e.g. the Czech Republic, Russia, Romania, Bulgaria) were frequently British nationals. Indeed, the UK was the third largest nationality of PLAB candidates (12%). Browsing internet forums used by medical school applicants, such as The Student Room [12], confirms widespread interest among would-be medical students in these medical schools and success in admission. Given McManus’ notion of ‘the academic backbone’, in which earlier academic attainment predicts later academic attainment [13], it would be unsurprising that applicants rejected from UK medical schools would perform worse in UK postgraduate examinations than those who achieved entry to medicine in the UK. Indeed, we found that UK national candidates required significantly more attempts to pass PLAB Part 1 than those of other nationalities. The first-attempt score on PLAB Part 1 was also significantly lower for UK nationals than non‐UK nationals [2].

The ambivalence of PMQ-to-background attribution is clearly important, though probably a small threat to the findings of studies such as that of Mehdizadeh et al. Researchers must know the nationality of doctors as well as their country of PMQ.

## Conclusion: future directions

Simplistic stereotypical analyses of doctors’ behaviour are no longer credible. In the UK, until 1991, English-qualified doctors were most often struck off the Medical Register for sexual shenanigans, while Scottish and Irish-qualified doctors were most frequently struck off for alcohol-related activities. The number of overseas-qualified doctors was then relatively small [14].

Mehdizadeh et al. contribute substantially to our understanding of the predictors (or concomitants) for review of doctors under the GMC’s performance procedures. Differences in the prevalence of referral for PAs by country of PMQ appear supported by quite separate evidence from the MRCGP examinations.

Using qualitative research approaches, explanations should examine the frequently-repeated concerns that unconscious bias “prevalent through much of the NHS,” leading to “disparate treatment of international medical graduates,” may have contributed to differential PA referral rates [6].

Future work could include PA outcomes and pay attention to the nationalities of those referred by the regulator, as well as their country of PMQ. Those who study the detailed results of postgraduate examinations might suggest that the training institutions of qualification, rather than just the country, should form the unit of aggregation in such research. Within its public datasets the GMC should at least include country of PMQ and nationality in its overarching summaries of postgraduate examination performance of all doctors in training.
